# The Digital Ageing Atlas: integrating the diversity of age-related changes into a unified resource

**DOI:** 10.1093/nar/gku843

**Published:** 2014-09-17

**Authors:** Thomas Craig, Chris Smelick, Robi Tacutu, Daniel Wuttke, Shona H. Wood, Henry Stanley, Georges Janssens, Ekaterina Savitskaya, Alexey Moskalev, Robert Arking, João Pedro de Magalhães

**Affiliations:** 1 Integrative Genomics of Ageing Group, Institute of Integrative Biology, University of Liverpool, Liverpool, UK; 2 University of North Carolina at Chapel Hill, NC, USA; 3 Skolkovo Institute of Science and Technology, Moscow region, Russia; 4 Institute of Biology of Komi Science Center of RAS, Syktyvkar, Russia; 5 Moscow Institute of Physics and Technology, Dolgoprudny, Russia; 6 Department of Biological Sciences, Wayne State University, Detroit, MI, USA

## Abstract

Multiple studies characterizing the human ageing phenotype have been conducted for decades. However, there is no centralized resource in which data on multiple age-related changes are collated. Currently, researchers must consult several sources, including primary publications, in order to obtain age-related data at various levels. To address this and facilitate integrative, system-level studies of ageing we developed the Digital Ageing Atlas (DAA). The DAA is a one-stop collection of human age-related data covering different biological levels (molecular, cellular, physiological, psychological and pathological) that is freely available online (http://ageing-map.org/). Each of the >3000 age-related changes is associated with a specific tissue and has its own page displaying a variety of information, including at least one reference. Age-related changes can also be linked to each other in hierarchical trees to represent different types of relationships. In addition, we developed an intuitive and user-friendly interface that allows searching, browsing and retrieving information in an integrated and interactive fashion. Overall, the DAA offers a new approach to systemizing ageing resources, providing a manually-curated and readily accessible source of age-related changes.

## INTRODUCTION

Ageing can be defined as a progressive functional decline, or a gradual deterioration of physiological function with age, often including a decrease in fecundity (1). Human ageing is characterized by multiple changes at different levels of biological organization (2,3). It is still not clear which (if any) molecular, cellular or physiological changes are more important drivers of the process of ageing or how they influence each other. One difficulty in understanding how different processes at different scales relate to ageing as a whole is the lack of integrative, holistic views of ageing. This hinders studies of how different molecular, cellular and physiological components interact with each other, in spite of the recognized importance of such approaches (4,5).

Particularly now in the post-genome era, efforts to obtain a more comprehensive and detailed characterization of molecular changes with ageing, such as those using -omics approaches (6–8), have been widespread. Use of this quantitative data, including its meta- and re-analysis, allows the application of systems biology approaches to ageing research. Consequently, there is now a drive to link these molecular level changes to cellular and physiological processes. The ultimate aim is to elucidate how molecular changes with age, for example, may influence or are influenced by changes in the wider organism, e.g. hormonal changes, and ultimately how these interactions contribute to pathology. Nonetheless, collating and converting raw data into information that is usable and can be cross compared is time consuming and difficult. In this context, we developed the Digital Ageing Atlas (DAA; http://ageing-map.org/), the first portal encompassing age-related changes at different biological levels, including a large amount of -omics data, already processed, categorized and filtered for statistical significance.

## DIGITAL AGEING ATLAS CONTENT, INTERFACE AND STRUCTURE

Conceptually, an age-related change represents an observed difference of a molecule, parameter or process between young and old, and various and diverse types of properties can be represented in a quantitative and/or qualitative way. To catalogue and organize age-related changes, in the DAA they fall into four broad categories: molecular, physiological, psychological and pathological changes (Table 1). The DAA contains: more than 3000 molecular ageing changes, which include gene expression, epigenetic and proteomic changes; over 300 physiological changes, which include cellular, hormonal and changes at various scales (including organs and the whole organism); and psychological or cognitive changes. Also included are pathological changes, listing epidemiological data on the incidence and/or mortality of major age-related diseases. Our focus is on changes occurring during normal ageing across populations, though e.g. gender-specific changes are indicated. As detailed below, data was manually-curated from the literature, such as textbooks and papers, and retrieved from public databases like GEO (9). All changes are fully referenced making it possible to access the raw data. In total, the DAA currently details 3526 biological changes in humans and 713 changes in mice. The DAA focuses on human data, however mouse data has been included, in particular gene expression data, and cross-linked to relevant human entries (e.g., homologous genes), to enhance and expand the information on human ageing. We anticipate the addition of data from other model organisms in the future.

**Table 1.** The number of human age-related changes for each category in the Digital Ageing Atlas

Type of change	Description	Number of changes
Molecular	Changes with age of a molecular nature, most being gene-centric	3071 (2599 genes)
Physiological	All non-molecular physiological ageing changes	343
Psychological	Cognitive and behaviour changes with age	17
Pathological	Changes in disease incidence or mortality for age-related diseases	95

**Figure 1.**
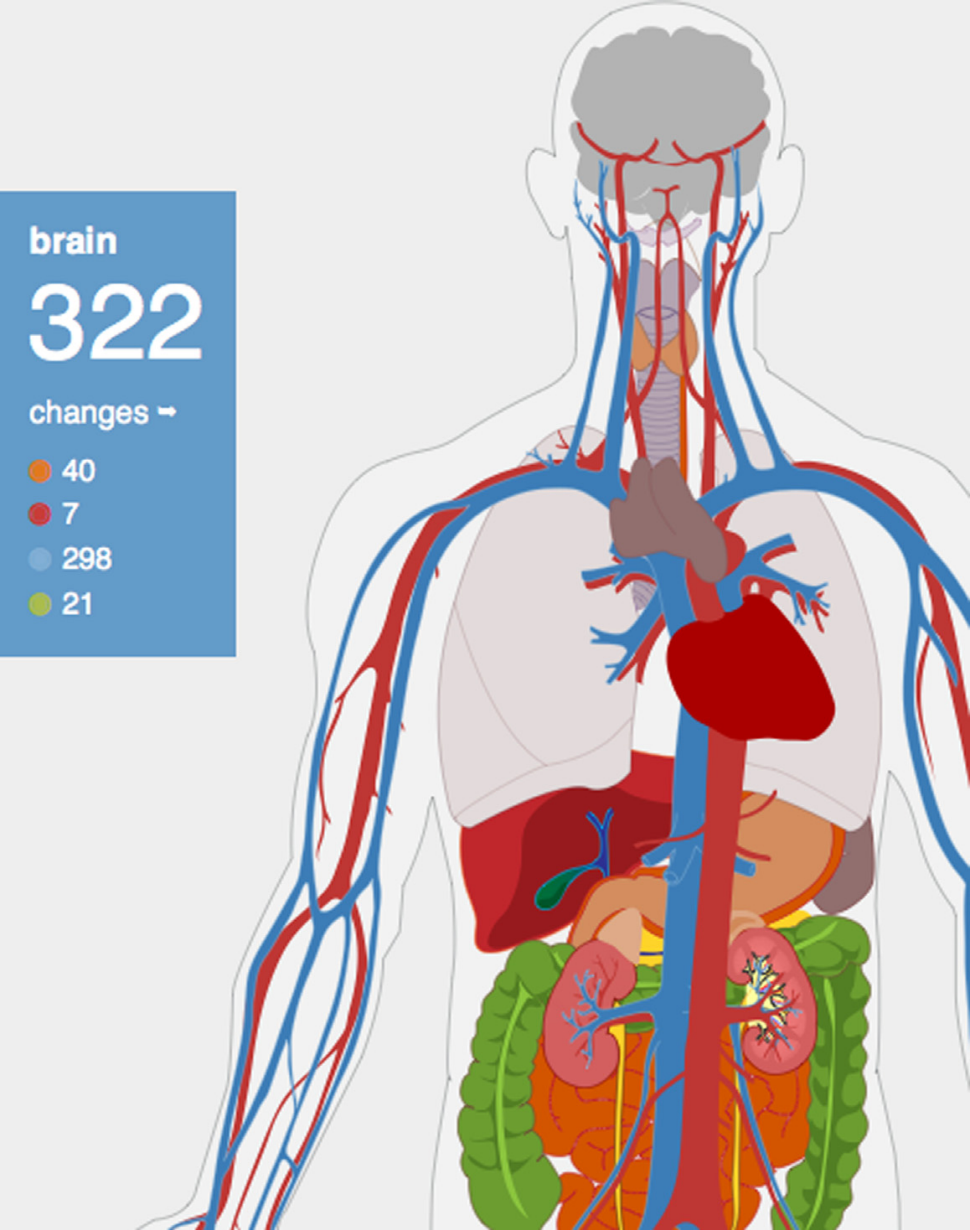
The DAA anatomical model. Moving the mouse over a given organ reveals the number of age-related changes in the DAA, along with a breakdown of the number of each specific type of change. Colours indicate the number of changes for each change type (orange: physiological, red: pathological, blue: molecular, green: psychological).

Presenting information in an easy-to-understand visual form is a powerful means of fostering the analysis and interpretation of large datasets and of allowing researchers to identify gaps in knowledge and develop new research directions (10,11). Without it the comprehension of large-scale or diverse datasets is impeded. Therefore, not only does the DAA merge different types of data into a single repository, but we developed an intuitive and user-friendly web resource that allows accessing, searching, browsing and retrieving the datasets in an integrated and interactive fashion. Specifically, we developed an anatomical diagram to allow users to browse and select their organ of interest (Figure 1). The use of keyword term searching (e.g. ‘heart’ will show both tissues and changes associated with the heart while ‘p53’ will show changes related to any gene with p53 in its name or alias) and more general anatomical selection offers a great deal of flexibility to users, ensuring that users of any level of technical skill can access the resources, including non-researchers, opening up the field of ageing to a wider audience.

**Figure 2.**
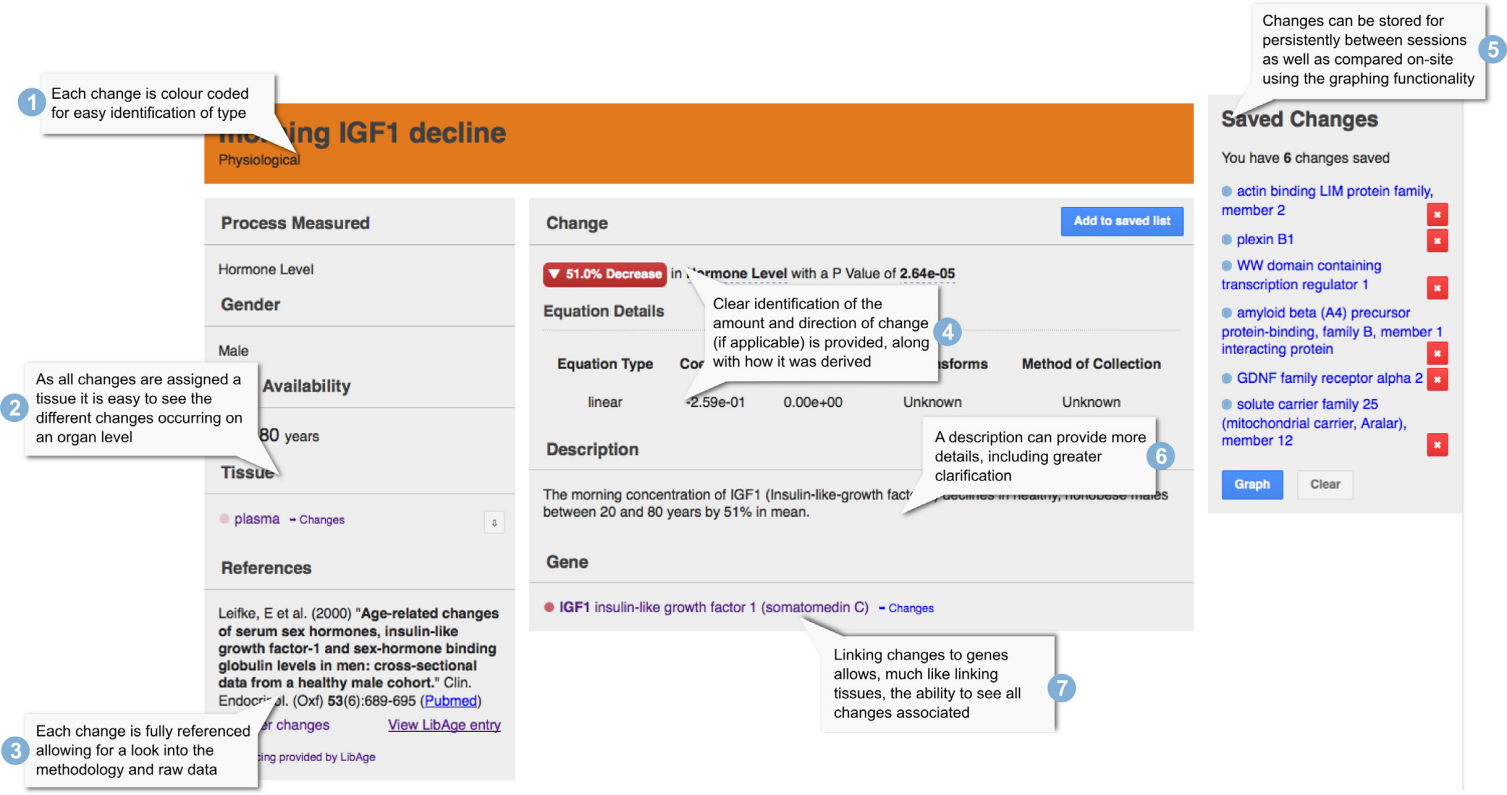
A labelled diagram of the entry for IGF1 age-related changes in the plasma: (1) Each change is colour coded for easy identification of type. (2) As all changes are assigned to a tissue it is easy to see the different changes occurring on an organ level. (3) Each change is fully referenced allowing for additional details into the methodology and access to the original data. (4) Clear identification of the amount and direction of change with age (if applicable) is provided, along with how it was derived. (5) Changes can be stored persistently between sessions as well as compared on-site using the graphing functionality. (6) Descriptions provide more details, including greater clarification regarding the context in which the change was observed and/or measured. (7) Linking changes to genes allows, much like linking tissues, the ability to see all the changes associated with a particular gene.

**Figure 3.**
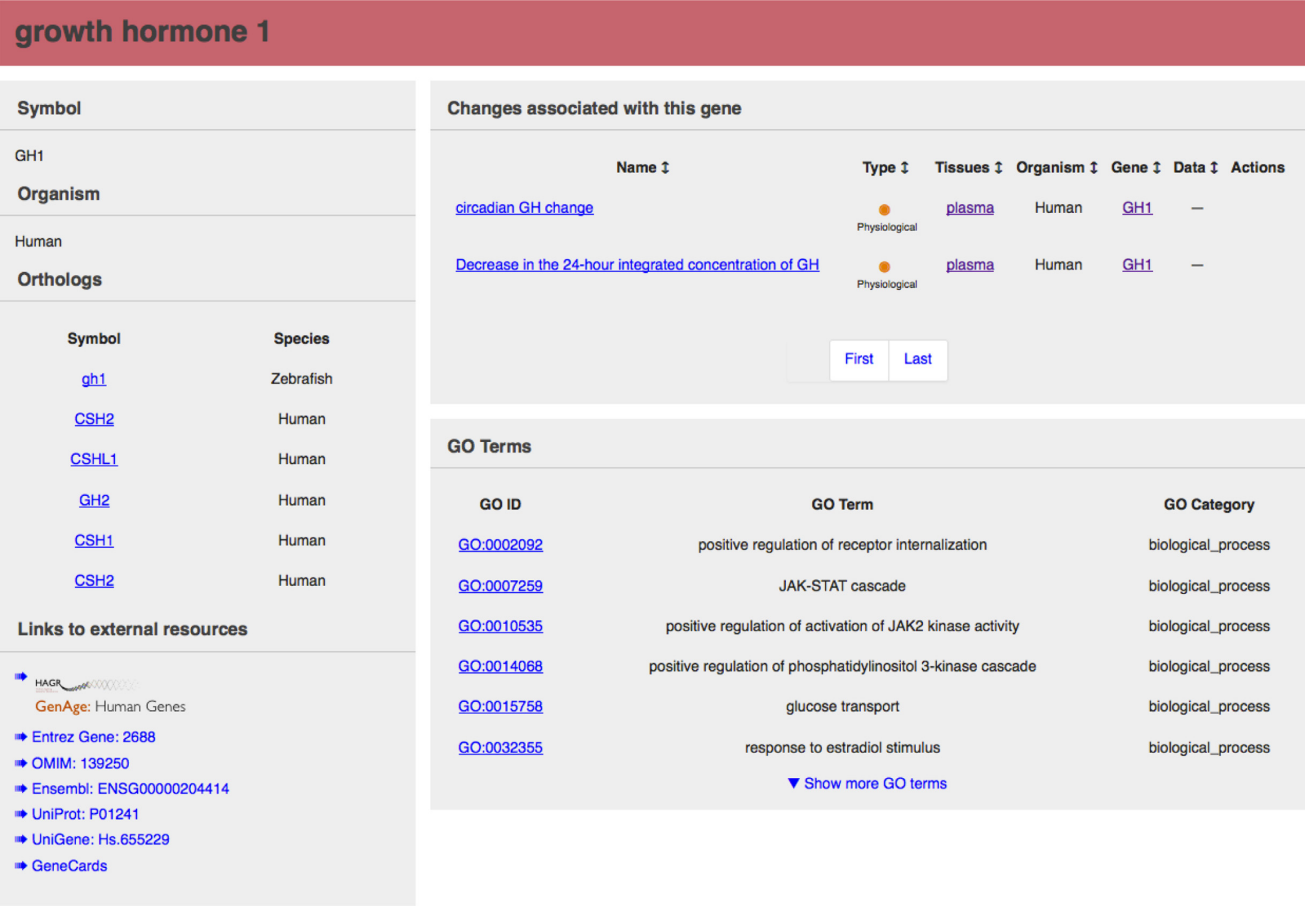
The details page for the gene *GH1*. This shows the two ageing changes associated with it and the links to external resources including GO terms, orthologs and various other databases.

Each age-related change in the DAA has its own page displaying a variety of information. Typically, entries include a description of the change with age, a quantification (if available) of the change with age (e.g. a percentage gene expression change between two ages), at least one reference and relevant links (Figure 2). The way in which the changes are stored in the database is best described in an object-orientated way. The key objects in the DAA are change, tissue, gene, property and data. The change object stores the basic information on a change including type, age of occurrence, gender (if available) and organism. The gene object contains basic information on a gene, e.g. symbol and name, mapping to other information such as homologues in other organisms, Gene Ontology (GO) terms and links to external resources, for instance cross-linking to the GenAge database of ageing-related genes (12) (Figure 3). Gene information can then be associated with multiple changes to prevent repetition and ensure ease of updating when elements such as the gene symbol change. It also allows for the DAA to display all changes associated with a gene making it easier to find information. The tissue object contains details on a tissue such as a name and description. The tissue objects (currently 284 different tissues are represented) are arranged into a simple hierarchical structure, based upon the ontology created by eVOContology (13), supplemented by descriptive data from both Brenda (14) and Wikipedia (http://en.wikipedia.org) and further expanded in our lab. Each tissue has a parent and zero or more children. The root parent represents the whole organism and the tissue hierarchy can be navigated on our interface. Each change is associated with one or more tissues, allowing for exploration of the number and types of changes occurring in each tissue or organ.

The property object allows for non-duplicate properties to be defined and associated with changes (e.g. the location property may take values like synapse, mitochondria, cellular, etc). These properties are defined by the curators and can encompass any value which may be relevant to the change but is not recorded elsewhere. This allows for great flexibility in recording type-specific information (e.g. sub-cellular location) and can be filtered against in the search interface. The data object allows the association of specific types of data with a change. It is divided into sub-objects that cover a class of data, such as percentage, equation or dataset. These store specific sets of data with some fields such as ‘change measured’ common between all. Percentage objects store a simple percentage value; equation objects store the components of an equation describing the change in a quantitative fashion; dataset objects store an arbitrary number of data points to plot more fragmentary data such as mortality rates. With this the database can cover most types of data that can be associated with a change during the life course, and more can be easily added, if required, with very little effort. These data are presented on the details page and used for the display of increase/decrease icons on the search page, among others.

The relationship object stands alone as it is not directly related to a change. Instead, similar to the tissue object, it stores a hierarchical representation of information. Each relationship object is linked to a single change and optionally another relationship object. These are then chained together to create a tree. Multiple trees can be associated with a change and can describe different types of associations from causal relationships to similar processes that are occurring together. Linking the relationship objects to changes allows the construction of complex hierarchies often encompassing different biological levels while still permitting a given change to appear in multiple hierarchies. A change can appear in multiple trees as the change may be a part of multiple processes, some of which may not be closely related. A good example of this is DAA982 (which refers to changes in CD16 expression in the elderly) in which there are two trees describing how the gene (a molecular change) reacts during two distinct physiological ageing changes. Relationships also link pathologies to physiological and molecular changes associated with them, like for Alzheimer's disease (DAA615) which is associated with neuritic plaques (DAA723) and beta-amyloid deposits (DAA1996).

**Figure 4.**
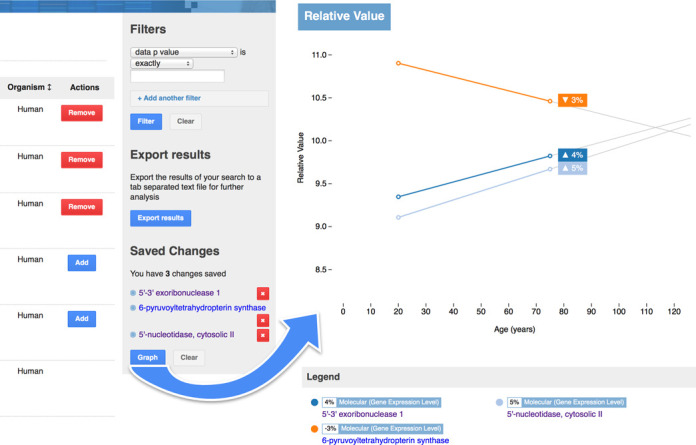
Storing changes for later analysis. A combination of two screenshots showing how changes can be added to the saved list and then compared against each other using the graphing capabilities of the Digital Ageing Atlas. Filters allow for a narrowing of results based on the properties of each change; Multiple filters can be applied. The actions column provides the ability to add and remove changes to the stored list.

Interpretation and visualization of the data is facilitated by tools built into the DAA. Any numerical change within the DAA can be compared against others by adding them to a list which can then be analysed in a graph form within the website. For example, a comparison can be made of molecular changes presented in graph form allowing the comparison of the gene expression levels recorded by those changes (Figure 4). More complex analyses can be performed using external tools as the DAA permits downloading both through its export tool and through the availability of the complete DAA dataset for download. The export tool itself takes advantage of the search and filtering supported and allows for specific subsets of data to be extracted and saved as a tab-delimited text file. The DAA and all its data is made available under a permissive licence.

## DATA SELECTION AND CURATION

Data in the DAA is manually curated and each age-related change has been selected based upon clearly defined criteria. First, only age-related changes for which there is direct, empirical evidence supported by one or more references are included. Second, only ageing changes occurring *in vivo* are incorporated into the DAA. Since our goal is to define typical age-related changes, we focused on those observed during healthy ageing, with the obvious exception of pathological age-related changes that describe mortality and incidence rates of specific diseases of the aged. Although the goal is to make the DAA as complete as possible, the focus is on what are likely the most important age-related changes, which in many cases are the changes that are also involved in determining age-related pathologies. Negative results can be included if these are deemed relevant to understand ageing, e.g. DAA711 refers to measurements of heart physiology that are unchanged with age. Our general policy regarding conflicting reports is to cite all conflicting reports and let users make their own decisions on how to interpret them.

Molecular changes (e.g. gene expression, protein levels and methylation) from high-throughput approaches are usually selected based on criteria for statistical significance that the authors have used in the sourced data, though data and methods (e.g. correction for multiple hypothesis testing) are examined as part of our QC procedures which are described in de Magalhães *et al*. (6). A *P*-value cut-off of 0.001 or lower is normally used for genome-wide approaches. This value was reached based on standard practice and observation of effects of the data and ensures that the changes added are above the noise threshold. Ours is an inclusive policy, however, and effect sizes and *P*-values are included in the DAA to allow users to make their own decisions about which data is relevant. The primary sources of data for the molecular section have been the meta-analysis by de Magalhães *et al*. (6), public databases like GEO (9) and primary publications. Therefore, quantitative data can be taken directly from publications or recomputed as in (6) with the specific type of equation used indicated in the details page for a given entry. At the time of writing the DAA includes 24 datasets from high throughput screens (mainly microarrays) that cover 22 different tissues.

Physiological changes were sourced from books (2,3,15), reviews and primary publications. Major changes in cell populations are likely to contribute to age-related physiological and pathological changes, therefore studies of cellular alterations with age are included, but results from *in vitro* cellular models of ageing are not included in the DAA. Pathological and some physiological changes were sourced from the Centers for Disease Control and Prevention (CDC) (http://www.cdc.gov/nchs/hdi.htm), selected based on their relevance to chronic ageing conditions and the ages which they cover. Psychological changes were sourced from the same locations as the physiological changes with the condition that they must indicate a change in behaviour or cognition as the organism ages.

Ageing changes vary between individuals and populations. It is not the goal of the DAA to capture the individual diversity of age-related changes and thus the relative dependence on large datasets. The objective of the DAA is to provide an overview of major age-related changes and so typical values are featured, though outliers are indicated in notes-specific to each data type. For example, gender-specific changes are featured and properly annotated. An attempt is made to obtain data from consistent sources. In mice, age-related changes and even lifespan can vary between strains, therefore the C57BL/6 strain is used as the ‘gold standard’ in the case of conflicting findings, however discrepancies are highlighted when they occur. The C57BL/6 strain was selected because, currently, it is the most commonly used mouse strain for ageing studies. This strategy is consistent with other similar projects like AGEMAP (16) and the Allen Brain Atlas (17) that also focus on the C57BL/6 strain. If age-related changes are suspected of being population-specific, then this is indicated in the DAA through a specific property.

The site itself uses the Python-based Django framework and is served by an Nginx web server. It uses PostgreSQL 9.1 as a database backend, implementing a number of constraints to ensure entries are not duplicated or left as orphans when a change instance is deleted. A web-based curation application was also created that allows for easy addition and updating of data without requiring knowledge of the technical operations of the portal. We encourage contributions by the wider research community. By having an intuitive and easily usable curation interface this provides the ability to both quickly correct and add relevant information as well as allowing specialists to directly contribute to its improvement, thus ensuring that it stays at the forefront of ageing research.

## AVAILABILITY

The DAA is available at http://ageing-map.org with the data made available under the permissive Creative Commons licence, allowing data to be used in other analyses. There are options to either download the entire database or to download more focused data using the export tool. Feedback via email is welcome, as are submissions of new data, for which a submission form is provided to ensure that the relevant information is included.

## CONCLUSION

The DAA is an integrated web resource for studying and visualizing human age-associated changes at various biological levels. It can aid researchers to perform integrative, system-level analysis of ageing. While target users are primarily fundamental researchers, it is anticipated that the DAA will also be useful to clinicians, students and the public in general. Other existing ageing-related resources such as GenAge (12), AgeFactDB (18) and SAGEWEB (http://sageweb.org/) focus on genes and factors that alter lifespan and/or ageing. By providing a manually-curated and readily accessible source of age-related changes during the normal life course, the DAA is thus complementary to existing resources and offers a new approach to systemizing ageing resources. This brings numerous benefits, limiting duplication of efforts and maintaining the accuracy of data which is essential given the rapid pace at which the field of ageing is progressing. In conclusion, the DAA aims to become the most comprehensive source for data related to ageing changes, consistently providing high-quality data, covering a wide variety of different biological levels.
